# Improving Image Quality in Medical Images Using a Combined Method of Undecimated Wavelet Transform and Wavelet Coefficient Mapping

**DOI:** 10.1155/2013/797924

**Published:** 2013-12-07

**Authors:** Du-Yih Tsai, Eri Matsuyama, Hsian-Min Chen

**Affiliations:** ^1^Department of Radiological Technology, Graduate School of Health Sciences, Niigata University, 2-746 Asahimachi-dori, Niigata 951-8518, Japan; ^2^Department of Biomedical Engineering, College of Engineering, Hungkuang University, Taichung 43302, Taiwan

## Abstract

We propose a method for improving image quality in medical images by using a wavelet-based approach. The proposed method integrates two components: image denoising and image enhancement. In the first component, a modified undecimated discrete wavelet transform is used to eliminate the noise. In the second component, a wavelet coefficient mapping function is applied to enhance the contrast of denoised images obtained from the first component. This methodology can be used not only as a means for improving visual quality of medical images but also as a preprocessing module for computer-aided detection/diagnosis systems to improve the performance of screening and detecting regions of interest in images. To confirm its superiority over existing state-of-the-art methods, the proposed method is experimentally evaluated via 30 mammograms and 20 chest radiographs. It is demonstrated that the proposed method can further improve the image quality of mammograms and chest radiographs, as compared to two other methods in the literature. These results reveal the effectiveness and superiority of the proposed method.

## 1. Introduction

Denoising and contrast enhancement operations are two of the most common and important techniques for medical image quality improvement. Because of their importance, there has been an enormous amount of research dedicated to the subject of noise removal and image enhancement [1–4].

With regard to image denoising, some approaches using discrete wavelet transform (DWT) have been proposed [5–7]. The DWT is very efficient from a computational point of view, but it is shift variant. Therefore, its denoising performance can change drastically if the starting position of the signal is shifted. In order to achieve shift invariance, researchers have proposed the undecimated DWT (UDWT) [8–10]. Mencattini et al. reported a UDWT-based method for the reduction of noise in mammographic images [11]. The reported method was robust and effective. However, the method was not advantageous in terms of computational aspects. Zhao et al. proposed an image denoising method based on Gaussian and non-Gaussian distribution assumptions for wavelet coefficients [12]. Huang et al. reported on a denoising method which involves directly selecting the thresholds for denoising by evaluating some statistical properties of the noise [13]. Recently, Matsuyama et al. proposed a modified UDWT approach to mammographic denoising [14]. The results demonstrated that the method could further improve image quality and decrease image processing time.

As regard to the improvement of contrast enhancement, various image enhancement techniques have been proposed [15–20]. These techniques can be divided into several categories, including histogram equalization [15, 16], region-based [17], fuzzy [18], genetic algorithm [19], and adaptive methodology [20]. Wavelet-based approaches to enhancement of digital images have been also reported [21–25]. Tsai et al. proposed a method which employs an exponential-type mapping function to the wavelet coefficients of digital chest images and then reconstructs an enhanced image with the mapped wavelet coefficients [22, 23]. Lee et al. used a sigmoid-type mapping function for wavelet coefficient weighting adjustment to improve the contrast of medical images [25]. The method was applied to chest radiographs, mammograms, and chest CT images. The method showed a statistically significant superiority over the exponential-type mapping function.

In this study, we expanded upon the previously suggested modified UDWT method [14] and combined it with the sigmoid-type mapping function [25]. By combining the two methods together in sequence, an effective algorithm for both image denoising and enhancement could be obtained. Original images were first denoised using the modified UDWT, followed by image enhancement using the wavelet coefficient mapping function. Finally, a denoised and contrast enhanced image was reconstructed by the inverse wavelet transform. In this study, we investigated the effectiveness of the proposed method by comparing it with two methods in the literature [14, 25].

## 2. Methods and Materials

### 2.1. Combined Method of Undecimated Discrete Wavelet Transform and Wavelet Coefficient Mapping

**Figure 1:**
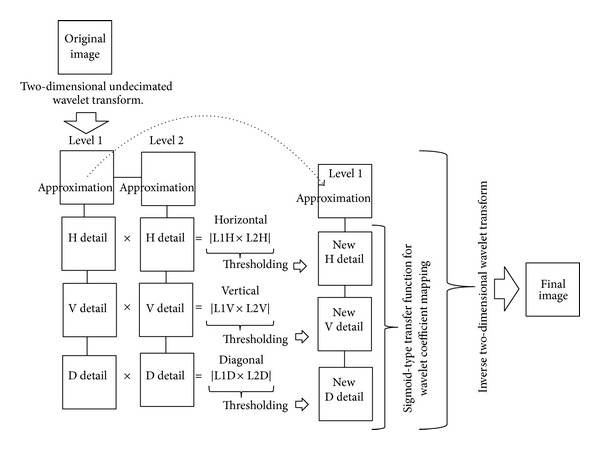
A flowchart summarizing the processing procedure for the proposed algorithm.

Figure 1 shows a flowchart of our proposed method. In the first phase, denoising was applied to original images using our newly adopted UDWT. In the second phase, image enhancement was performed using a sigmoid-type transfer function for wavelet coefficient mapping. Sections 2.1.1 and 2.1.2 describe the two phases of the proposed method, respectively.

#### 2.1.1. Extended Undecimated Discrete Wavelet Transform Method

The UDWT is a wavelet transform algorithm designed to overcome the lack of translation invariance of the DWT. Unlike the DWT, the UDWT does not incorporate the downsampling operations. Thus, the approximation coefficients (low-frequency coefficients) and detailed coefficients (high-frequency coefficients) at each level are the same length as the original signal. The basic algorithm of the conventional UDWT is that it applies the transform at each point of the image and saves the detailed coefficients and uses the approximation coefficients for the next level. The size of the coefficients array does not diminish from level to level. This decomposition operation is further iterated up to a higher level. There are major differences between the modified UDWT method [14] and the conventional UDWT method. First, the conventional UDWT decomposes the original image (level 0) into one low-frequency band and three high-frequency bands for each resolution level with the same size as the original image. The decompositions are usually conducted up to resolution level 4. In contrast, the modified UDWT method only needs to perform the computation up to resolution level 2 and repeat the computation only one time [14, 26]. Second, the conventional UDWT thresholded the detailed coefficients at all 4 levels with the same thresholding value, while the modified UDWT method utilizes the hierarchical correlation of the coefficients between level 1 and level 2 of the three detailed coefficients for thresholding. In other words, the thresholding values vary and are dependent on the nature of the noise.

The extended UDWT method adopted in the present study was based on the modified UDWT [14]. The method we used mainly consisted of the following steps (see Figure 1).

(1) Perform two-dimensional UDWT to the original image to obtain wavelet coefficients up to level 2.
(2) Calculate the hierarchical correlations of the detailed coefficients between level 1 and level 2 for the three subbands. The correlations for the three detailed subbands are given as
  (1) $$\begin{array}{c} \left|{\text{Coef}}_{\text{lev}\_1}\left(p,q\right)\times {\text{Coef}}_{\text{lev}\_2}\left(p,q\right)\right|, \end{array}$$
  where *p* and *q* are the new coordinates after wavelet transform. Coef_lev_1_ and Coef_lev_2_ are wavelet coefficients of level 1 and level 2, respectively.
(3) Determine threshold values for each detailed subband. The determination procedure is as follows
  (a) Generate a correlation image Img_Cor_(*p*, *q*) for each detailed subband:
    (2) ImgCor(p,q) =|Coeflev1(p,q)×Coeflev2(p,q)|.
  (b) Find the maximum value in each row in the horizontal (*x*-) direction of the obtained correlation image for each of the three detailed subbands.
  (c) Compute the mean of the maximum values obtained from all rows in the *x*-direction of the correlation image. The mean is denoted by Mean_max⁡_
  (d) Eliminate those correlation values greater than 0.8 × Mean_max⁡_. These excluded values are considered signal data. The value of 0.8 was determined empirically through experiments.
  (e) Compute the standard deviation *σ* from the remaining correlation values.
  (f) Determine the threshold value by use of the following formula:
    (3) $$\begin{array}{c} \text{THR}=1.6\times \sigma . \end{array}$$
    The value of 1.6 was determined empirically through experiments.
(4) Apply the determined threshold values to the correlation values:
  (4) New  Coeflev_1(p,q)={Coeflev_1(p,q),if  |Coeflev_1×Coeflev_2|≥THR,0,otherwise,
  where New  Coef_lev_1_(*p*, *q*) is the newly obtained, modified coefficient for level 1. The modified coefficients of the horizontal, vertical, and diagonal subbands are, respectively, obtained. It should be noted that the threshold operation was only applied to the detailed components. The reason is that the detailed components mainly contain the noise and high-frequency information, whereas the approximation component that mainly contains low-frequency information remains unchanged.
(5) Perform the inverse wavelet transform to reconstruct the denoised image with the approximation coefficient of level 1 and the three newly obtained detailed coefficients of level 1.

In a previous study [14], we evaluated six comparatively popular wavelet basis functions, namely, discrete FIR approximation of Meyer wavelet (dmey), Daubechies order 2 (db2), Symlets order 7 (sym7), Coiflets order 1 (coif1), Coiflets order 5 (coif5), and biorthogonal 6.8 (bior6.8), as candidates for selection as the most suitable basis function for the UDWT. The evaluation results showed that wavelet-processed images with db2 basis function provided the best results among the six basis functions. Thus, we selected db2 basis function for the proposed method [14, 25, 26].

#### 2.1.2. Wavelet Coefficient Mapping

A sigmoid-type transfer curve with a one-to-one mapping function was used for enhancement of image contrast [25]. The mapping function was determined based on the following considerations: (a) wavelet coefficients having high values are heavily weighted because they carry more useful information; (b) the coefficients at low levels are heavily weighted because they carry detailed information, such as edge information; and (c) the approximation coefficients are not manipulated to prevent image distortion [23, 25].

The input coefficient *w*_input_^*j*^(*m*, *n*) of level *j* at position (*m*, *n*) was manipulated using the sigmoid-type transfer curves of wavelet coefficients. The mapping function is given by

(5) woutputj(m,n) =a×11+{1/exp⁡[(winputj(m,n)−c)/b]}  ×winputj(m,n),

where *w*_output_^*j*^(*m*, *n*) represents output coefficient.  *a*,  *b*, and *c* are constants and are determined depending on the extent of enhancement to be added. In practice, (6) is used as the mapping function instead of (5). In (6), the values of the coefficients are expressed in terms of percentage for the ease of computation:

(6) woutputj=a×11+[1/exp⁡((winputj−c)/b)]×winputj [%].

Here, *w*_input_^*j*^ is the input value expressed in terms of percentage. This value makes the mean of the absolute values of the coefficients at level *j* equal to 50%. Notation *w*_output_^*j*^ is the corresponding output value expressed in terms of percentage. By utilization of percentage, the constants  *a*,  *b*, and *c* could be used independent of image characteristics. The value of constant  *a*  was obtained using (7):

(7) $$\begin{array}{c} a=2-\frac{\left(j-1\right)}{N}, \end{array}$$

where *N* represents the maximum decomposition level. Consequently, the lower the wavelet decomposition level  *j*, the greater the gradient of the transfer curve becomes. As a result, the wavelet coefficients at low-decomposition levels that contain information about edges of an image are highly weighted. The constant *c* was determined by use of (8):

(8) $$\begin{array}{c} c=d+b\times \log_{e}\left(a-1.0\right), \end{array}$$

where *d* is a constant used for determining the inflection point of the sigmoid curve, and *b* represents a constant used for determining the gradient of the sigmoid curve. The values of *b* and *d* used in this study were 20 and 25, respectively [23].

### 2.2. Image Data

To evaluate and validate our proposed method, we used two standard digital databases: a mammogram database and a chest radiograph database. The former was from the Mammographic Image Analysis Society (MIAS) [27] and the latter was from the Japanese Society of Radiological Technology [28]. Patient informed consent was not required. A total of 30 mammograms obtained from the database were used for investigation of the effectiveness of the proposed method. The matrix size of each image was 1024 × 1024 pixels with 8-bit gray-level resolution. The matrix size of each chest radiograph was 2048 × 2048 pixels with 12-bit gray-level resolution. From the radiograph database, 20 chest images were used for the present study.

Other than the described image data, we also prepared another data set by purposely adding a zero-mean Gaussian noise with a standard deviation of 0.01 to the obtained 30 mammograms and 20 chest radiographs. The purpose of using the images with external added noise was to clearly demonstrate the effectiveness of the proposed method by comparing the pixel-value profiles along the horizontal direction of processed images. As for visual perceptual evaluation, in order to keep visual evaluation clinically practical, images without adding external noise were used for visual assessment.

### 2.3. Visual Perceptual Evaluation

A visual perceptual evaluation was designed for performance analysis. We used Scheffe's method of paired comparison to evaluate the preference of overall image quality [29, 30]. The visual evaluation was made by five experienced radiological technologists (ranging from 20 to 25 years of experience). In the case of mammograms, the obtained 30 mammograms from the data set were processed using the proposed method, the modified UDWT method, and the sigmoid-type wavelet coefficient mapping method. Thus, a total of 90 images were used for image quality evaluation. All images were evaluated on a pair of widely used medical 3 M monochrome liquid-crystal display monitors. Each observer reviewed the images independently. The reading time was limited to less than 20 seconds for each reading. The observers independently evaluated one pair of images, which were shown on the monitors one at a time, using a 5-point grading scale (−2 points to +2 points). If the image shown on the left was much better than that shown on the right in terms of overall image quality, the left image was given +2 points; the left image was given +1 point when it was slightly better than the right one; the left image was given 0 points, when both images were of the same image quality. Conversely, if the image shown on the left was much poorer than that shown on the right in terms of overall image quality, the left image was given −2 points; the left image was given −1 point when it was slightly poorer than the right one. Comparisons were made by use of three possible combinations, that is, modified UDWT/sigmoid mapping, modified UDWT/proposed method, and sigmoid mapping/proposed method combinations. Each pair of images was determined randomly. In addition, the two paired images (left side versus right side) were arranged on a random basis.

The same procedures were performed for the case of chest radiographs.

### 2.4. Quantitative Evaluation

In order to compare objectively the performance of the proposed algorithm against two published algorithms [14, 25], in this study we adopted four image quality metrics. The 4 metrics are the mean-to-standard-deviation (MSR), the contrast to noise ratio (CNR), contrast improvement ratio (CIR), and peak signal-to-noise ratio (PSNR). They are briefly described as follows.

The MSR [31, 32] in a desired region of interest (DROI) is defined as

(9) $$\begin{array}{c} \text{MSR}=\frac{\mu_{d}}{\sigma_{d}}, \end{array}$$

where *μ*_*d*_ and *σ*_*d*_ are the mean and standard deviation computed in the DROI. The CNR [31, 32] is defined as

(10) $$\begin{array}{c} \text{CNR}=\frac{\left|\mu_{d}-\mu_{\mu }\right|}{\sqrt{0.5\left(\sigma_{d}^{2}+\sigma_{\mu }^{2}\right)}}, \end{array}$$

where *μ*_*μ*_ and *σ*_*μ*_ are the mean and the standard deviation computed in an undesired region of interest (UROI) such as background. Both the MSR and CNR measurements are proportional to the medical image quality.

The CIR [33] is a quantitative measurement of the contrast improvement and is defined as

(11) $$\begin{array}{c} \text{CIR}=\frac{\sum_{i}\sum_{j}\left|c\left(i,j\right)-c^{\prime }{\left(i,j\right)}^{2}\right|}{\sum_{i}\sum_{j}c{\left(i,j\right)}^{2}}, \end{array}$$

where *c*(*i*, *j*) and *c*′(*i*, *j*) are the local contrast values of original and enhanced images, respectively. The local contrast *c*(*i*, *j*) is defined by the difference of mean values in two rectangular windows centered on a pixel at the coordinate (*i*, *j*). In detail the *c*(*i*, *j*) is given by

(12) $$\begin{array}{c} c\left(i,j\right)=\frac{\left|p\left(i,j\right)-a\left(i,j\right)\right|}{\left|p\left(i,j\right)+a\left(i,j\right)\right|}, \end{array}$$

where *p* and *a* are the average values of pixels within a 3 × 3 region and a 7 × 7 surrounding neighborhood, respectively. The greater the CIR value, the better the enhancement result.

The PSNR [34] in decibels is adopted for measuring the performance of denoising and is given by

(13) $$\begin{array}{c} \text{PSNR}=10\;\text{lo}{\text{g}}_{10}\frac{M\times N\times T^{2}}{\sum_{i}\sum_{j}{\left[d\left(i,j\right)-d^{\prime }\left(i,j\right)\right]}^{2}}, \end{array}$$

where *M* × *N* is the size of the image, *T* ^2^ is the maximum possible value that can be obtained by the image signal, *d*(*i*, *j*) and *d*′(*i*, *j*) are the pixel-values of original and processed images, respectively. The higher the PSNR value, the better the performance of denoising.

## 3. Results

In this study, we used 30 mammograms and 20 chest radiographs to evaluate the proposed method by comparing it to two other existing methods: a modified UDWT method [14] and a sigmoid-type wavelet coefficient (STWC) mapping method [25]. The results of a previous study showed that by use of a modified UDWT method the computation time can be reduced to approximately 1/10 that of the conventional UDWT method. In addition, the results of visual assessment indicated that the images processed with the modified UDWT method showed statistically significant superior image quality over those processed with the conventional UDWT method [14]. The STWC mapping method demonstrated that it offers considerably improved enhancement capability as compared to the conventional enhancement methods, such as the fast Fourier transform method, the conventional wavelet-based method, and the conventional exponential-type wavelet coefficient mapping method [25].

**Figure 2:**
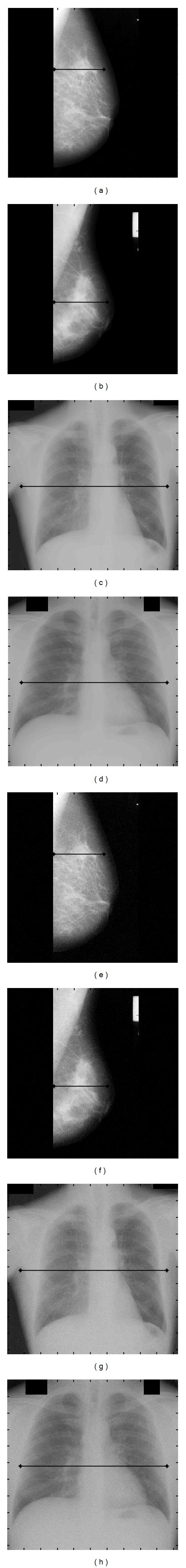
Examples of images used for this study. Images shown in the upper row are original images: (a) and (b) are two mammograms and (c) and (d) are two chest radiographs. The corresponding images are in the lower row with external added noise.

**Figure 3:**
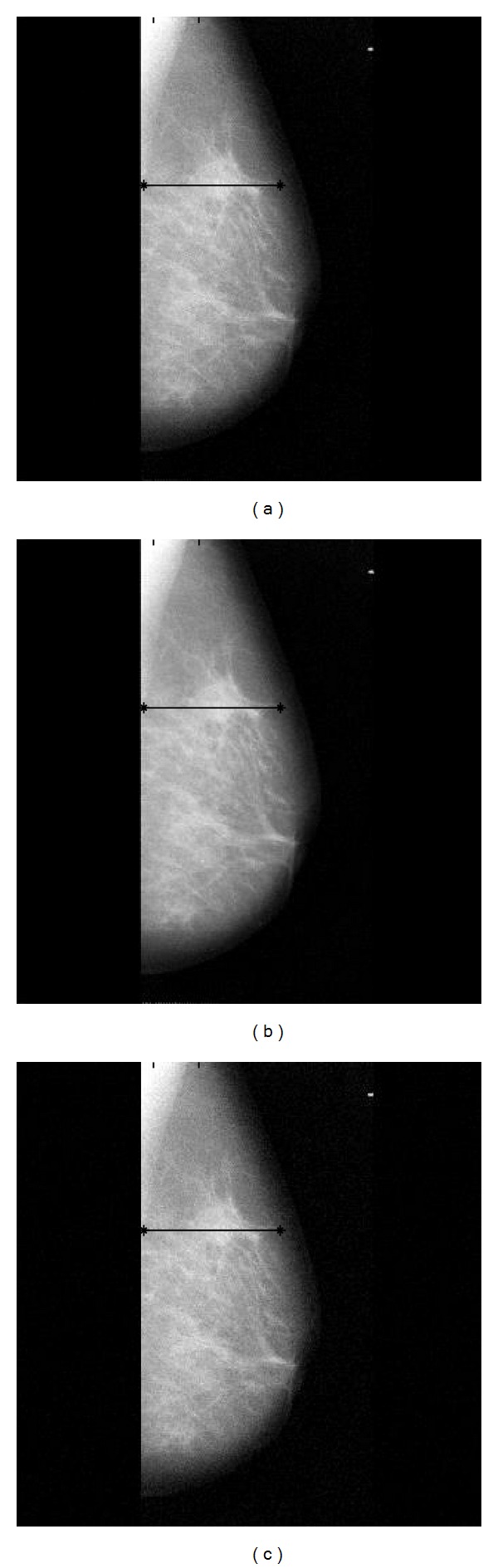
Image processing results for mammograms. (a) Image processed by the proposed method, (b) image processed by the modified UDWT method, and (c) image processed by the sigmoid-type wavelet coefficient mapping method.

Figure 2 shows two sets of example images of mammograms and chest radiographs. Original images are shown in the upper row of the figure and corresponding images are shown in the lower row with external noise added.  Figure 3 illustrates an example of image processing results obtained from the mammogram shown in Figure 2(e). Figures 3(a), 3(b), and 3(c) are resulting images processed by using the proposed method, the modified UDWT method, and the STWC mapping method, respectively.

**Figure 4:**
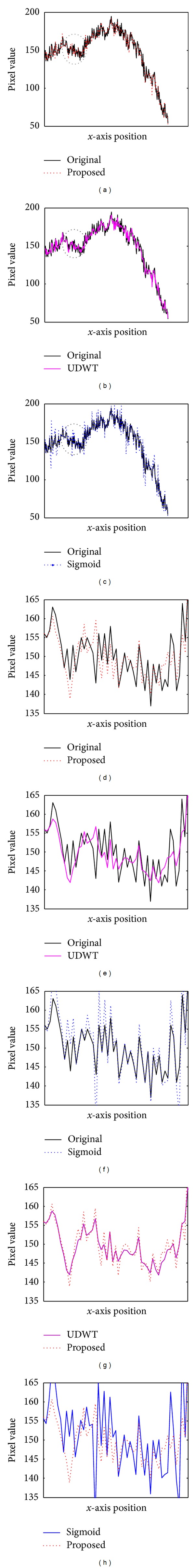
An example showing pixel-value profiles from original and processed mammograms. (a)–(c) Original versus processed by the proposed method, the modified UDWT method, and the sigmoid-type wavelet coefficient mapping method, respectively. The profiles were measured along the horizontal lines (black lines) as shown in Figures 3(a)–3(c). (d)–(f) Corresponding magnified profiles indicated by circles as shown in (a)–(c), respectively. (g) Profiles of two processed images; the solid line indicates the profile of an image processed by the modified UDWT method, and the dotted line indicates that by the proposed method. (h) Profiles of two processed images; the solid line indicates the profile of an image processed by the sigmoid-type wavelet coefficient mapping method, and the dotted line indicates that by the proposed method.

Figure 4 shows the *x-*direction profiles of the processed images traced from the lines indicated on the images of Figures 3(a)–3(c). Figures 4(a)–4(c) illustrate the profiles of the images processed by the proposed method, the modified UDWT method, and STWC mapping method, respectively. The *x*-direction profile of the original image traced from the line indicated on the image of Figure 2(e) is also shown in the figures for comparison. Figures 4(d)–4(f) show the magnified views of the profiles corresponding to the positions indicated by the dotted circles in Figures 4(a)–4(c), respectively. The pixel-value profile of the image obtained with the proposed method and that of the image obtained with the modified UDWT method are shown in Figure 4(g). The pixel-value profile of the image obtained with the proposed method and that of the image obtained with the STWC mapping method are shown in Figure 4(h). It is obvious from Figure 4(g) that the pixel-value profile of the image processed by the proposed method is much more enhanced at the edges than that of the image processed by the modified UDWT method. It is also apparent from Figure 4(h) that the noise has been significantly reduced by employing the proposed method.

**Figure 5:**
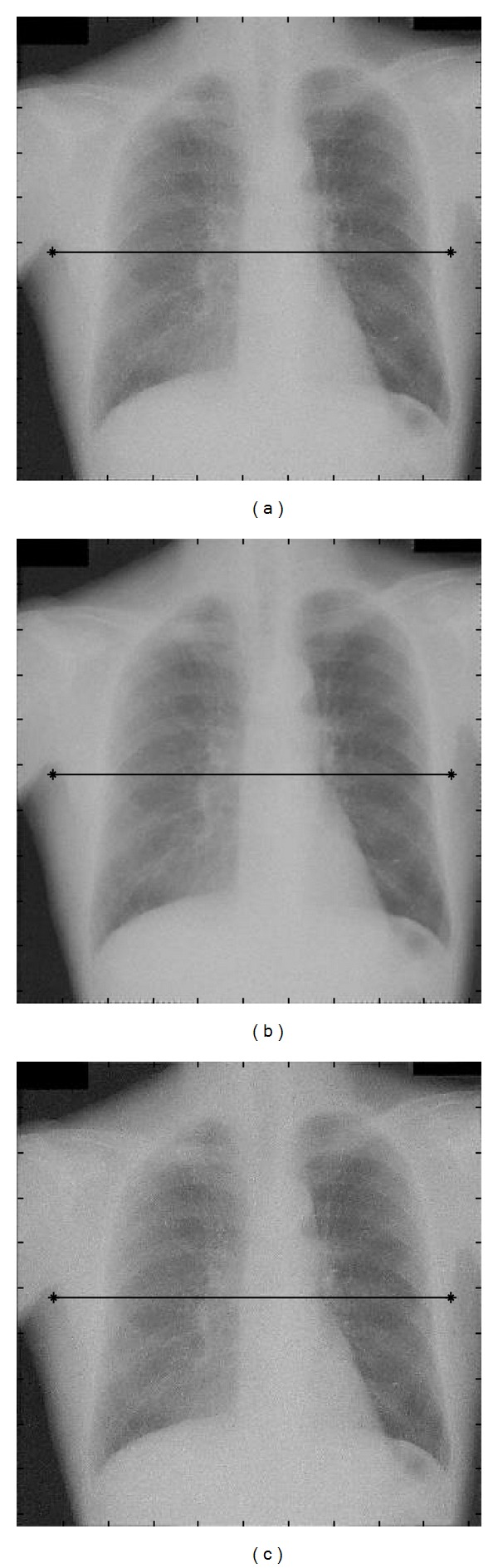
Image processing results for chest radiographs. (a) Image processed by the proposed method, (b) image processed by the modified UDWT method, and (c) image processed by the sigmoid-type wavelet coefficient mapping method.

Similarly, Figure 5 illustrates an example of image processing obtained from the chest radiograph shown in Figure 2(g). Figures 5(a), 5(b), and 5(c) are resulting images processed by using the proposed method, the modified UDWT method, and the STWC mapping method, respectively.

**Figure 6:**
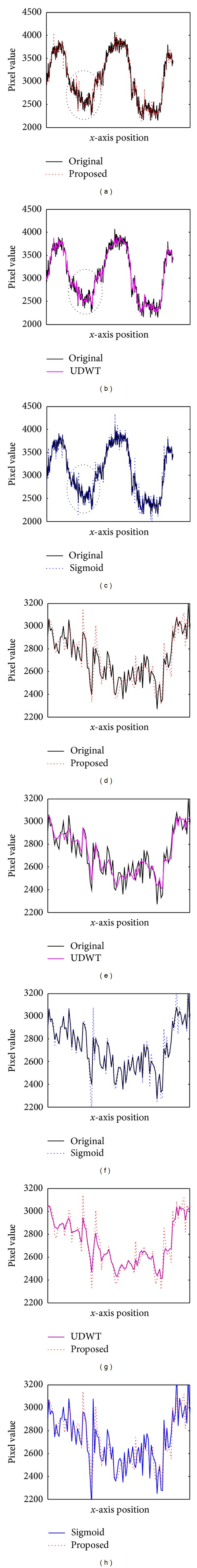
An example showing pixel-value profiles from original and processed chest radiographs. (a)–(c) Original versus processed by the proposed method, the modified UDWT method, and the sigmoid-type wavelet coefficient mapping method, respectively. The profiles were measured along the horizontal lines (black lines) as shown in Figures 5(a)–5(c). (d)–(f) Corresponding magnified profiles indicated by circles as shown in (a)–(c), respectively. (g) Profiles of two processed images; the solid line indicates the profile of an image processed by the modified UDWT method, and the dotted line indicates that by the proposed method. (h) Profiles of two processed images; the solid line indicates the profile of an image processed by the sigmoid-type wavelet coefficient mapping method, and the dotted line indicates that by the proposed method.

Figure 6 shows the *x*-direction profiles of the processed images traced from the lines indicated on the images of Figures 5(a)–5(c). Figures 6(a)–6(c) illustrate the profiles of the images processed by the proposed method, the modified UDWT method, and the STWC mapping method, respectively. The *x*-direction profile of the original image traced from the line indicated on the image of Figure 2(g) is also shown in the figures for comparison. Figures 6(d)-6(f) show the magnified views of the profiles corresponding to the positions indicated by the dotted circles in Figures 6(a)–6(c). The pixel-value profile of the image obtained with the proposed method and that of the image obtained with the modified UDWT method are shown in Figure 6(g). The pixel-value profile of the image obtained with the proposed method and that of the image obtained with the STWC mapping method are shown in Figure 6(h). It is obvious from Figure 6(g) that the pixel-value profile of the image processed by the proposed method is much more enhanced at the edges than that of the image processed by the modified UDWT method. It is also apparent from Figure 6(h) that the noise has been significantly reduced by employing the proposed method.

**Figure 7:**
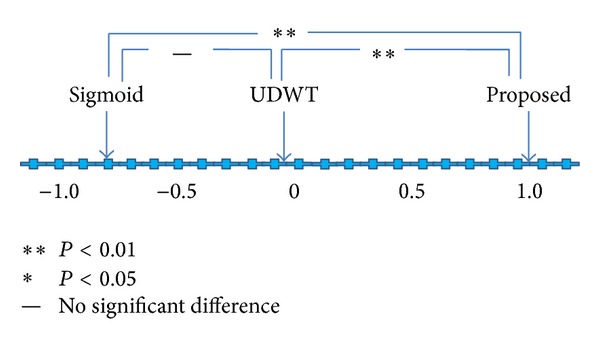
Preference ranking map for the three image groups: STWC-mapping-method-processed, modified-UDWT-processed, and proposed-method-processed mammograms.

The results of scoring for the three combinations by the five observers are listed in Tables 1 and 2 for mammograms and chest radiographs, respectively. As described earlier, if the left image of the paired images (two-image combination) was poorer than the right image in terms of overall image quality, it received a negative score.  Table 1 summarizes the visual results for the case of mammograms. As indicated by the preference scores shown in the rightmost column of the table, the images processed by the proposed method were judged to have the best quality.  Figure 7 illustrates visual evaluation results using Scheffe's method of paired comparisons. The results are shown by a preference ranking map for the three image groups, namely, the proposed method, the modified UDWT method, and the STWC mapping method. The figures shown on the horizontal line of the map are average preference degrees of the three groups. The average preference degrees were obtained from the average main effects by use of the data shown in Table 1. The images processed by the proposed method show the highest ranking, followed by those processed by the modified UDWT method and those processed by the STWC mapping method. A two-tailed  *F*  test was used to measure statistical significance. The difference between the processed images of the proposed method and those of the modified UDWT method was statistically significant (*P* < 0.05). The difference between the processed images of the proposed method and those of the STWC mapping method was also statistically significant (*P* < 0.01). However, there was no significant difference between the processed images of the modified UDWT method and those of the STWC mapping method.

**Figure 8:**
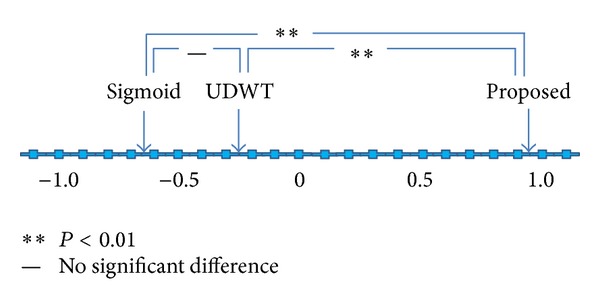
Preference ranking map for the three image groups: STWC-mapping-method-processed, modified-UDWT-processed, and proposed-method-processed chest radiographs.

Table 2 summarizes the visual results for the case of chest radiographs. As shown in the rightmost column of the table, the images processed by the proposed method were judged to have the best quality.  Figure 8 shows visual evaluation results using Scheffe's method of paired comparisons. As shown in the figure, the images processed by the proposed method show the highest ranking, followed by those processed by the modified UDWT method and those processed by the STWC mapping method. The difference between the processed images of the proposed method and those of the modified UDWT method and the difference between the processed images of the proposed method and those of the STWC mapping method were statistically significant (*P* < 0.01). However, there was no significant difference between the processed images of the modified UDWT method and those of the STWC mapping method.

Tables 3 and 4 summarize the quantitative evaluation results for the proposed method and two published methods in terms of MSR, CNR, CIR, and PSNR metrics. As described in Section 2.4 the MSR and CNR measurements are proportional to the medical image quality. It is obvious from the tables that both MSR and CNR values of the images processed by the proposed method give the best results as compared to those processed by the other two methods. The CIR is a metric used for evaluating the contrast improvement. It is noted from the results shown in Tables 3 and 4 that the proposed method shows the greatest value, followed by the sigmoid mapping and modified UDWT. The reason why the proposed method is superior to the sigmoid mapping method is due to the fact that the images processed by the proposed method have been denoised prior to mapping operation. In the case of PSNR measurement, the results listed in the tables show that the modified UDWT method was slightly better than both the proposed method and sigmoid mapping method from the point of view of denoising performance. The reason might be because some residual (unremoved) noise has also enhanced at enhancement operation. This results in the decrease of PSNR value. However, the images processed by the proposed method showed the best overall image quality in terms of both denoising and contrast enhancement when looking into the values of the MSR and CNR as shown in Tables 3 and 4.

## 4. Discussion and Conclusion

In this study, we proposed an algorithm which combines the modified UDWT method and the sigmoid-type wavelet coefficient mapping method. The results of visual evaluation, as illustrated in Figures 7 and 8, suggested that the proposed method was significantly superior to the two previously reported methods. It is apparent from Figures 4(g) and 4(h) and Figures 6(g) and 6(h) that the proposed method combines the advantages of the two methods: denoising and contrast enhancement. The results of the quantitative evaluation also showed that the proposed method outperformed over the two other methods.

By using our proposed method, the computation time can be reduced to 2 seconds (personal computer, DELL, OPTIPLEX 960), approximately 1/10 of the computing time compared to the conventional UDWT method. The reason for enabling reduction of processing time lies in the following fact: in the conventional UDWT method, the decomposition and composition processes are usually conducted up to resolution level 4. That is, the method needs to process a total of 12 images (3 detailed coefficients for each of the 4-resolution levels) for wavelet transforms and inverse transforms and it results in time consumption. In contrast, the proposed method only needs to perform the process up to resolution level 2 and repeat the calculation one time. Therefore, only 6 images (3 detailed components for each of the 2-resolution levels) were required for processing. As a result, the computing time using the proposed method can be much reduced.

This study has several limitations. First, we only applied the proposed method to mammograms and chest radiographs. In order to validate the versatility of the proposed algorithm, application of the proposed method to other images obtained from different modalities, such as ultrasound, digital radiography, and SPECT is needed. Second, the value shown in (3) used for determining threshold value and that shown in (8) used for determining the gradient of the sigmoid curve were empirically selected. A method for automated selection is desirable. Finally, our dataset contained only 30 mammograms and 20 chest radiographs. A larger dataset may enable us to better evaluate the performance of the proposed method.

In summary, we proposed a method for improving image quality in medical images by using a wavelet-based approach. The proposed method integrated two components: image denoising and image enhancement. In the first component, a modified undecimated discrete wavelet transform was used to eliminate the noise. In the second component, a wavelet coefficient mapping function was applied to enhance the contrast of denoised images obtained from the first component. We examined the performance of the proposed method by comparing it with two previously reported methods. The results of visual assessment indicated that the images processed by the proposed UDWT method showed statistically significant superior image quality over the other two methods. The results of quantitative assessment also showed that the proposed UDWT method outperformed over the two other methods. Our research results demonstrated the superiority and effectiveness of the proposed method. This methodology can be used not only as a means for improving visual quality of medical images but also as a preprocessing module for computer-aided detection/diagnosis systems to improve the performance of screening and detecting regions of interest in images.

## Figures and Tables

**Table 1:** Results of mammogram scoring for the three combinations by the five observers.

Combination		Observer					
		a	b	c	d	e	Sum
Sigmoid	UDWT	−1.1	−0.87	0	−1.2	−1.2	−4.37
Sigmoid	Proposed	−1.57	−1.4	−1.67	−1.47	−1.6	−7.71
UDWT	Proposed	−1.33	−1.27	−1.47	−1.3	−1.5	−6.87

**Table 2:** Results of chest radiograph scoring for the three combinations by the five observers.

Combination		Observer					
		a	b	c	d	e	Sum
Sigmoid	UDWT	−1	−0.4	−0.1	−0.95	−1.25	−3.7
Sigmoid	Proposed	−1.7	−1.4	−1.35	−1.4	−1.65	−7.5
UDWT	Proposed	−1.5	−1.35	−1.5	−1.4	−1.55	−7.3

**Table 3:** Comparison of image processing methods in terms of 4 quantitative quality metrics for mammograms.

Method	MSR	CNR	CIR	PSNR
UDWT	5.80	8.18	0.28	38.35
Sigmoid	6.09	7.64	0.29	36.39
Proposed	6.24	8.24	0.67	37.98

**Table 4:** Comparison of image processing methods in terms of 4 quantitative quality metrics for chest radiographs.

Method	MSR	CNR	CIR	PSNR
UDWT	6.36	2.21	0.46	37.61
Sigmoid	6.32	2.17	0.53	36.66
Proposed	6.46	2.29	0.71	36.76

## References

[1] Mencattini A, Salmeri M, Lojacono R, Frigerio M, Caselli F (2008). Mammographic images enhancement and denoising for breast cancer detection using dyadic wavelet processing. *IEEE Transactions on Instrumentation and Measurement*.

[2] Scharcanski J, Jung CR (2006). Denoising and enhancing digital mammographic images for visual screening. *Computerized Medical Imaging and Graphics*.

[3] Tsai D-Y, Lee Y, Chiba R An improved adaptive neighborhood contrast enhancement method for medical images.

[4] Yoon B-W, Song W-J (2007). Image contrast enhancement based on the generalized histogram. *Journal of Electronic Imaging*.

[5] Fodor IK, Kamath C (2003). Denoising through wavelet shrinkage: an empirical study. *Journal of Electronic Imaging*.

[6] Ferreira CBR, Borges DL (2003). Analysis of mammogram classification using a wavelet transform decomposition. *Pattern Recognition Letters*.

[7] Cho D, Bui TD, Chen G (2009). Image denoising based on wavelet shrinkage using neighbor and level dependency. *International Journal of Wavelets, Multiresolution and Information Processing*.

[8] Fowler JE (2005). The redundant discrete wavelet transform and additive noise. *IEEE Signal Processing Letters*.

[9] Starck J-L, Fadili J, Murtagh F (2007). The undecimated wavelet decomposition and its reconstruction. *IEEE Transactions on Image Processing*.

[10] Wang X-Y, Yang H-Y, Fu Z-K (2010). A new wavelet-based image denoising using undecimated discrete wavelet transform and least squares support vector machine. *Expert Systems with Applications*.

[11] Mencattini A, Rabottino G, Salmeri M, Lojacono R, Sciunzi B (2010). Denoising and enhancement of mammographic images under the assumption of heteroscedastic additive noise by an optimal subband thresholding. *International Journal of Wavelets, Multiresolution and Information Processing*.

[12] Zhao P, Shang Z, Zhao C (2012). Image denoising based on Gaussian and non-Gaussian assumption. *International Journal of Wavelets, Multiresolution and Information Processing*.

[13] Huang Z, Fang B, He X, Xia L (2009). Image denoising based on the dyadic wavelet transform and improved threshold. *International Journal of Wavelets, Multiresolution and Information Processing*.

[14] Matsuyama E, Tsai D-Y, Lee Y (2013). A modified undecimated discrete wavelet transform based approach to mammographic image denoising. *Journal of Digital Imaging*.

[15] Kim W, You J, Jeong J (2012). Contrast enhancement using histogram equalization based on logarithmic mapping. *Optical Engineering*.

[16] Papadopoulos A, Fotiadis DI, Costaridou L (2008). Improvement of microcalcification cluster detection in mammography utilizing image enhancement techniques. *Computers in Biology and Medicine*.

[17] Rangayyan RM, Shen L, Shen Y (1997). Improvement of sensitivity of breast cancer diagnosis with adaptive neighborhood contrast enhancement of mammograms. *IEEE Transactions on Information Technology in Biomedicine*.

[18] Jiang J, Yao B, Wason AM (2005). Integration of fuzzy logic and structure tensor towards mammogram contrast enhancement. *Computerized Medical Imaging and Graphics*.

[19] Hashemi S, Kiani S, Noroozi N, Moghaddam ME (2010). An image contrast enhancement method based on genetic algorithm. *Pattern Recognition Letters*.

[20] Tsai D-Y, Lee Y, Chiba R An improved adaptive neighborhood contrast enhancement method for medical images.

[21] Strickland RN, Hahn H (1996). Wavelet transforms for detecting microcalcifications in mammograms. *IEEE Transactions on Medical Imaging*.

[22] Tsai D-Y, Lee Y, Sakaguchi S (2002). A preliminary study of wavelet-coefficient transfer curves for the edge enhancement of medical images. *Transactions of the Japanese Society for Medical and Biological Engineering*.

[23] Tsai D-Y, Lee Y A method of medical image enhancement using wavelet-coefficient mapping functions.

[24] Heinlein P, Drexl J, Schneider W (2003). Integrated wavelets for enhancement of microcalcifications in digital mammography. *IEEE Transactions on Medical Imaging*.

[25] Lee Y, Tsai D-Y, Suzuki T (2008). Contrast enhancement of medical images using sigmoid-type transfer curves for wavelet coefficient weighting adjustment. *Medical Imaging and Information Science*.

[26] Matsuyama E, Tsai DY, Lee Y, Takahashi N Comparison of a discrete wavelet transform method and a modified undecimated discrete wavelet transform method for denoising of mammograms.

[27] http://peipa.essex.ac.uk/info/mias.html.

[28] http://www.jsrt.or.jp/jsrt-db/eng.php.

[29] Scheffe H (1959). *The Analysis of Variance*.

[30] Canavos GC, Koutrouvelis JA (2008). *An Introduction to the Design & Analysis of Experiments*.

[31] Bao P, Zhang L (2003). Noise reduction for magnetic resonance images via adaptive multiscale products thresholding. *IEEE Transactions on Medical Imaging*.

[32] Cincotti G, Loi G, Pappalardo M (2001). Frequency decomposition and compounding of ultrasound medical images with wavelet packets. *IEEE Transactions on Medical Imaging*.

[33] Wang Y-P, Wu Q, Castleman KR, Xiong Z (2003). Chromosome image enhancement using multiscale differential operators. *IEEE Transactions on Medical Imaging*.

[34] Luisier F, Blu T, Unser M (2007). A new SURE approach to image denoising: interscale orthonormal wavelet thresholding. *IEEE Transactions on Image Processing*.

